# "If You Have to Ask, You'll Never Know": Effects of Specialised Stylistic Expertise on Predictive Processing of Music

**DOI:** 10.1371/journal.pone.0163584

**Published:** 2016-10-12

**Authors:** Niels Chr. Hansen, Peter Vuust, Marcus Pearce

**Affiliations:** 1 Center for Music in the Brain, Department of Clinical Medicine, Aarhus University & The Royal Academy of Music Aarhus/Aalborg, Aarhus, Denmark; 2 School of Communication and Culture, Aarhus University, Aarhus, Denmark; 3 Cognitive Science Research Group and Centre for Digital Music, Queen Mary, University of London, London, United Kingdom; 4 Cognitive and Systematic Musicology Laboratory, School of Music, Ohio State University, Columbus, OH, United States of America; University of Zurich, SWITZERLAND

## Abstract

Musical expertise entails meticulous stylistic specialisation and enculturation. Even so, research on musical training effects has focused on generalised comparisons between musicians and non-musicians, and cross-cultural work addressing specialised expertise has traded cultural specificity and sensitivity for other methodological limitations. This study aimed to experimentally dissociate the effects of specialised stylistic training and general musical expertise on the perception of melodies. Non-musicians and professional musicians specialising in classical music or jazz listened to sampled renditions of saxophone solos improvised by Charlie Parker in the bebop style. Ratings of explicit uncertainty and expectedness for different continuations of each melodic excerpt were collected. An information-theoretic model of expectation enabled selection of stimuli affording highly certain continuations in the bebop style, but highly uncertain continuations in the context of general tonal expectations, and vice versa. The results showed that expert musicians have acquired probabilistic characteristics of music influencing their experience of expectedness and predictive uncertainty. While classical musicians had internalised key aspects of the bebop style implicitly, only jazz musicians’ explicit uncertainty ratings reflected the computational estimates, and jazz-specific expertise modulated the relationship between explicit and inferred uncertainty data. In spite of this, there was no evidence that non-musicians and classical musicians used a stylistically irrelevant cognitive model of general tonal music providing support for the theory of cognitive firewalls between stylistic models in predictive processing of music.

## 1. Introduction

“If you have to ask, you’ll never know”. American jazz musician Louis Armstrong’s famous reply when prompted to define jazz unmistakably demonstrates how highly specialised expertise can be hard, and sometimes even undesirable, to capture in scientific terms. Although acquiring knowledge within specific musical styles constitutes a hallmark of expert musicianship, it remains to be determined which expertise-related enhancements of auditory processing (e.g. [1–6]) are ascribable to general musical training and exposure and which to specialised schooling and stylistic enculturation. This distinction is essential to exclude secondary factors, such as higher motivation for listening tasks in musicians [7] which may in itself lead to greater efficacy of auditory sequence learning [8].

The underrepresentation of genre-specific, specialised expertise in music cognition research [9] is evident from the prominence of categorical comparisons of musicians versus non-musicians within the behavioural sciences (e.g. [10]) and the neurosciences (e.g. [11]). Exceptions to this general pattern have typically adopted brain volumetrics or event-related potential techniques to demonstrate specialised training effects on key aspects of low-level, auditory perception, focusing primarily on instrument- rather than style-specific specialisation (e.g. [12–19]).

The probe-tone method, where listeners indicate on a rating scale the extent to which melodic continuations correspond to what they had expected [20, 21], offers a behavioural method of investigating stylistic expectations for higher levels of musical structure, both within and between cultures [22–29]. Taken together, studies using this paradigm suggest that melodic expectations of non-experts typically rely on salient surface features, such as pauses, register changes, and frequency of occurrence in the local probe-tone context, whereas those of enculturated experts are more dependent on abstract schemas relating to tonal hierarchies and global transitional probabilities within a given style of music [24–29]. These findings have been interpreted in terms of a general reliance on data-driven, bottom-up processing where expert listeners incorporate schema-driven, top-down processing to a greater extent than non-experts [29]. Others have argued that even apparent adherence to local features may be explained as surface manifestations of predictive top-down processing due to statistical prominence of specific features in musical styles [30, 31].

These findings confirm broader observations that predictive processing underlies numerous aspects of musical activities, including expressive performance timing [32], listening [33], interaction [34], improvisation [35], composition [36], and reading musical notation [4]. Because these skills are enhanced through practice and experience, expertise and stylistic specialisation, in particular, may ultimately be regarded as processes of predictive processing optimisation. Systematic studies of expectedness and uncertainty thus have wider societal implications, e.g. for educational practice and cross-cultural understanding.

Despite its clear merits, the probe-tone research summarised above also has limitations. First, given its cross-cultural emphasis, the widespread use of unfamiliar pitch material throughout these studies (e.g. Western participants listening to musical scales that are incompatible with the major/minor system) does not allow the findings to be generalised to situations where listeners have acquired stylistic models of more than one style. It has been proposed that “cognitive firewalls” exist between such models, restricting the use of acquired musical expectations by contextual relevance [37, 33]. Second, expert listeners’ veridical familiarity with specific pieces may confound schematic knowledge about the style. This could generate dichotomous rather than probabilistic continuation judgements in probe-tone studies [27, 28]. Third, lack of a systematic way of estimating and quantifying melodic expectation makes it hard to assess correspondence with schematic knowledge and distinguish probabilistic properties of different musical styles. Fourth, both behavioural and electrophysiological research has focused on expectedness, thus ignoring predictive uncertainty which may indeed constitute an important expertise-related top-down modulator of expectations [38].

The methodology of the present study directly countered these four limitations. First, bebop jazz was used to represent a musical style whose specialised listening grammar (cf. [39]) is highly constrained, albeit less familiar to the average Western listener [40, 41], despite belonging to the same cultural sphere and making use of well-known pitch material. Bebop evolved in North America during the 1940–1950s and is typically associated with musicians like Dizzy Gillespie, Charlie Christian, Thelonious Monk, Miles Davis, and Charlie Parker. While many scholars have documented an abundance of repeated melodic figures throughout bebop jazz [42–47], Charlie Parker’s improvisations have been found to be especially rich in motifs [43]. Motivic thinking is furthermore encouraged in jazz improvisation pedagogy [48, 49], leading to effortless transmission of melodic figures between interacting musicians [43]. This is particularly relevant for the present study because motifs entail low-entropy transitions which, in other styles, have been found to promote learnability [38].

Second, to address the potential confound of veridical familiarity, improvised solos were used because they are much less likely to have been internalised by expert musicians. Given that improvisation relies on real-time composition comprising uninterrupted, serial music making under strict time constraints [35], it provides a highly reliable manifestation of the bebop style as it is cognitively represented. Whereas previous studies have emphasised productive aspects with the explicit aim of modelling the cognitive processes underlying improvisation [35, 40, 41, 50–52], less is known about expert perception of such stimuli. Most probably, listening to improvised bebop solos requires highly specialised stylistic expertise while circumventing dichotomous expectations arising from veridical knowledge.

Third, the computational model Information Dynamics of Music (IDyOM) [53] was used to estimate probability distributions for every next pitch in a melody. This provided a way of quantifying and contrasting schematic expectations typical of bebop with more stylistically generalised ones. Four specific aspects of IDyOM enable us to acknowledge and capitalise upon previous findings while maintaining cognitive plausibility. First, successful replications of the bebop style from its inherent probabilistic properties [50–52, 54] call for the use of unsupervised statistical learning as implemented in IDyOM. Next, whereas previous studies have modelled bebop improvisation with zeroth-order [54] or higher-order, conditional statistics [50, 52], IDyOM accomplishes even greater predictive power by optimally combining Markov models of variable context lengths [53]. Moreover, whereas some previous modelling attempts have used distributions of pitch intervals rather than single scale degrees [51], none have so far combined the two, despite accumulating support for such an approach in modelling melodic expectations [38, 53]. The multiple viewpoint system implemented in IDyOM makes this possible [55]. Lastly, unlike IDyOM, previous probabilistic modelling of bebop has focused on objective complexity models of single pieces pieces [35, 56], rather than listeners’ dynamically changing subjective perception during listening. Combining the statistics of the local context with those of a large training dataset representative of a listener’s exposure history, IDyOM simulates more accurately the dynamic cognitive processes involved in musical listening.

Fourth, the present study embraced uncertainty as a key aspect of predictive processing which should be studied alongside confirmation and violation of expectations. Notably, when discussing and teaching improvisation, professional jazz musicians readily apply terms like “surprise”, “uncertainty”, “risk”, “novelty”, “complexity”, and “degrees of information” [56]. Aiming to model such musical uncertainty cognition, Hansen and Pearce [38] recently found that the Shannon entropy [57] of probability distributions estimated by IDyOM provided a good fit to listeners’ level of uncertainty when making predictions about melodic continuation in other genres. Musicians generally made predictions with lower uncertainty than non-musicians, particularly in contexts where statistical learning was enabled through low-entropy continuations, not unlike those found in idiomatic bebop motifs. However, as noted above, in contrast to IDyOM’s combination of long- and short-term expectations, previous jazz research has only relied on local entropy metrics derived from individual compositions to characterise improvisation [35, 56]. While we can presume that specialised bebop knowledge will be most advantageous when the statistics differ most strongly from general, common-practice tonal music, Hansen and Pearce’s [38] research design did not allow them to distinguish between the influence of generalised and specialised musical expertise.

The present study aimed to investigate the hallmarks left by specialised musical expertise on high-level predictive processing which distinguish it from generalised expertise or no expertise. In contrast to previous studies focusing on creative facets of bebop expertise [40, 41, 50–52], we address receptive aspects of specialisation within the style. Specifically, non-musicians and professional classical and jazz musicians listened to sampled renditions of improvised Charlie Parker solos while providing explicit uncertainty and expectedness ratings for different continuations of melodic excerpts. Stimuli afforded low- or high-entropy continuations within bebop (while simultaneously affording high- or low-entropy continuations, respectively, in the context of general tonal music). Comparing classical and jazz musicians enabled us to assess the influence of specialised expertise whereas comparing classical and non-musicians enabled us to assess the influence of generalised expertise.

## 2. Methods

### 2.1. Participants

Professional jazz musicians (*n* = 22; 4 females; median age = 32, IQR = 13), professional classical musicians (*n* = 20; 14 females; median age = 28, IQR = 7), and non-musicians (*n* = 20; 8 females; median age = 27.5, IQR: 15) were recruited for the study. Musicians were required to regularly perform concerts and/or contribute to commercial recordings, receiving the majority of their income from performing and/or teaching within the relevant musical genre. Full-time performance degree students in classical music or jazz were also eligible. Non-musicians must never have had regular one-on-one music lessons and not have performed music in public after the age of 12. Kruskall-Wallis and Chi-squared tests showed that the three groups were matched on age, *H(2)* = 3.764, *p* = .152, but not on gender, *χ*^*2*^(2) = 11.598, *p* = .003. Although the latter difference was considered irrelevant given that no studies have reported gender effects on melodic expectancy ratings, this represents a potential limitation that could be addressed in future research (see, for instance, [58], for possible gender differences in scalp topography).

Importantly, musicians scored significantly higher than non-musicians on the subscales and listening tests from *Goldsmiths Musical Sophistication Index* (Gold-MSI) [59] (Table 1). Gold-MSI constitutes a well-established measure of formal and informal engagement with musical activities, showing high internal consistency, high test-retest reliability, and high correspondence with other music-related self-report inventories and auditory musicality tests [59]. Whereas jazz and classical musicians were matched on all subscales, jazz musicians outperformed classical musicians on the genre sorting and melodic memory tests.

**Table 1:** Musical experience and listening skills.

	Gold-MSI: Self-report questionnaire						Gold-MSI: Tests		Jazz
	*F1*: *Active Engagement*	*F2*: *Perceptual Abilities*	*F3*: *Musical Training*	*F4*: *Singing Abilities*	*F5*: *Emotions*	*FG*: *General Musical Sophistication*	*Genre sorting (adj*. *Rand index)*	*Melodic memory (d’)*	*Composite jazz experience*
	M (SD)	M (SD)	M (SD)	M (SD)	M (SD)	M (SD)	M (SD)	M (SD)	M (SD)
**Jazz**	51.1 (6.5)	56.0 (*5*.*5*)	42.5 (4.7)	39.1 (5.4)	35.4 (4.3)	105.5 (10.2)	0.55 (0.1)	2.45 (0.7)	213 (18)
**Classical**	47.4 (8.3)	56.3 (*4*.*2*)	44.0 (2.2)	37.2 (5.7)	37.5 (2.9)	102.8 (10.1)	0.42 (0.2)	1.85 (0.6)	130 (15)
**Non**	34.5 (10.1)	42.1 (8.4)	10.6 (3.2)	24.8 (8.0)	30.2 (4.4)	57.3 (13.8)	0.38 (0.2)	0.77 (0.6)	99 (14)
**Jazz vs. classical**	*U* = 160, *p* = .13	*t*(40) = 0.19, *p* = .85	*U* = 206, *p* = .71	*t*(40) = -1.11, *p* = .28	*U* = 171, *p* = .21	*t*(40) = -0.86, *p* = .39	*t*(40) = -2.39, *p* = .02	*t*(40) = -2.79, *p* = .01	*t(40) = -16*.*38*, *p <* .*01*
**Musicians vs. non-musicians**	*U* = 108, *p* < .01	*U* = 66, *p* < .01	*U* = 0, *p* < .01	*t*(60) = 7.67, *p <* .01	*U* = 112, *p* < .01	*t*(60) = 15.16, *p <* .01	*U* = 289, *p* < .05	*t*(59) = 7.20, *p <* .01	*t*(54) = 9.75, *p* < .01

The table contains descriptive and inferential statistics concerning musical experience and listening skills for participants in the three participant groups. Means and standard deviations relate to scores on the subscales and listening tests from Goldsmiths Musical Sophistication Index (Gold-MSI) [59]. For statistical comparisons of jazz vs. classical musicians and musicians (i.e. jazz and classical) vs. non-musicians, independent-samples t-tests were used for normally distributed data whereas non-parametric Mann-Whitney U-tests were used for non-normally distributed data.

A composite measure of jazz-specific musical experience was obtained in the following way: (i) participants indicated the total number of jazz concerts performed and attended as well as the number of jazz songs in their personal music collection and active performance repertoire; (ii) all responses were reverse-scored by multiplying by -1; (iii) rank scores were computed for each of the four questions across all participants, with ties being assigned the maximum value (c.f. Modified Competition Ranking, “1334 rule”); (iv) the sum of ranks constituted each participant’s score. Unsurprisingly, jazz musicians had significantly greater jazz experience than classical musicians, and musicians also surpassed non-musicians (Table 1). Despite persistent recruitment efforts, the final sample of classical musicians had slightly higher jazz experience than non-musicians.

### 2.2. Stimuli

All stimuli originated from the untransposed C-instrument version of the *Omnibook* [60] containing transcribed solos by the American jazz saxophonist Charlie Parker (1920–1955). IDyOM, a computational model of expectation [53], was used to select 10 monophonic melodic contexts for each of the two conditions referred to as “high bebop entropy” and “low bebop entropy” (see S1 Text for details on stimulus selection). Only contexts from songs scoring low on familiarity (1 or 2 on a 5-point Likert scale), as judged by two independent jazz experts, were included.

**Fig 1:**
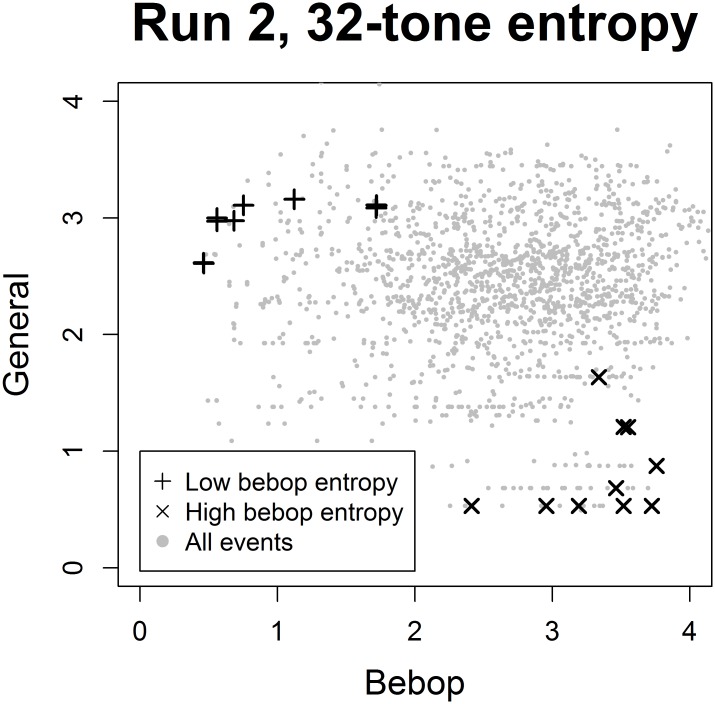
Entropy of final stimuli. Scatterplots of entropy estimates from the bebop and general models with the final stimuli marked with + (low bebop entropy) and ⨉ (high bebop entropy). The entropy estimates plotted here resulted from the second model run which pertained to the candidate contexts and were computed over the full distribution of the 32 pitches occurring in the Charlie Parker corpus (see Fig B in S1 Text for further details).

Stimuli in the high bebop entropy condition were selected to generate high uncertainty in an expert jazz listener about the next note according to a probabilistic model trained on the Charlie Parker solos (i.e. “bebop model”), but low uncertainty according to a model trained on Western choral and folk music (i.e. “general model”) (Table 2 in [31]). For a listener primarily exposed to common-practice tonal music, on the other hand, this same context would be expected to generate predictions with relatively low degrees of uncertainty. Conversely, low bebop entropy stimuli had low entropy according to the bebop model and high entropy according to the general model, presumably resulting in high-certainty expectations in jazz listeners and uncertain expectations in non-experts. The separation of stimuli used in the two conditions is evident from Fig 1.

**Table 2:** Expertise differences in bebop and general model-fit.

		Expectedness				Inferred uncertainty				Explicit uncertainty			
**Bebop**	**One-way ANOVA**	***F***	***df***	***p***	***η***^***2***^	***F***	***df***	***p***	***η***^***2***^	***F***	***df***	***p***	***η***^***2***^
	Expertise	9.42	2,59	.001***	0.24	2.98	2,59	.059	0.09	3.61	2,59	.033*	0.11
	**Planned contrasts**	***t***	***df***	***p***		***t***	***df***	***p***		***t***	***df***	***p***	
	Jazz vs. classical	1.66	59	.103		-0.69	38.0	.497		-2.17	59	.034*	
	Classical vs. non	2.60	59	.012*		-1.92	37.9	.063		-0.24	59	.808	
	Jazz vs. non	4.32	59	< .001***		-2.31	37.0	.026*		-2.42	59	.018*	
**General**	**One-way ANOVA**	***F***	***df***	***p***	***η***^***2***^	***F***	***df***	***p***	***η***^***2***^	***F***	***df***	***p***	***η***^***2***^
	Expertise	1.35	2,59	.268	0.04	3.00	2,59	.057	0.09	1.44	2,59	.246	0.05
	**Planned contrasts**	***t***	***df***	***p***		***t***	***df***	***p***		***t***	***df***	***p***	
	Jazz vs. classical	-1.08	36.8	.289		1.00	37.9	.322		1.48	59	.143	
	Classical vs. non	2.00	36.8	.053		1.59	37.9	.119		-0.05	59	.958	
	Jazz vs. non	0.44	33.4	.664		2.36	36.9	.023*		1.43	59	.158	

* *p* < .050,

** *p* < .010,

*** *p* < .001.

MIDI files were exported from Sibelius 7.1.3 (Avid Technology Inc.) using an alto saxophone sound. Prior to this, internal pauses were overwritten by extending the previous duration to prevent participants from responding before hearing the entire context. To ensure unambiguous pitch perception, stimuli were presented at half their original tempo and note durations of probe tones were further doubled. To increase ecological validity, a swing feel was added through linear interpolation from plots of amateur musicians’ preferred swing ratios recorded at five different tempi [61].

### 2.3. Procedure

The experiment comprised two phases completed while seated in a test booth wearing headphones for ~90 mins in total depending on individual pace and voluntary breaks. Before each phase, a trial melody was played with the experimenter present, and an opportunity was given to ask clarifying questions and adjust the sound level.

In Phase 1, melodic contexts were presented in randomized order without probe tones. After each trial, participants provided ratings of explicit certainty on a 9-point Likert scale using the numeric keys of the computer keyboard (1: “highly uncertain”; 9: “highly certain”). By default, liking ratings were also provided by the stimulus presentation software, but these were not included in the present analysis. Before each trial, a harmonic cadence, comprising a progression of three chords (ii7-V7-Imaj7) which establish an unambiguous sense of musical key and meter, was played with piano sounds. Participants were told that these cadences represented “filler sounds” to break up the contexts and were encouraged to use the full range of the rating scale.

Phase 2 comprised trials for nine different probe-tone continuations of each context presented in random order with participants providing expectedness ratings on a 9-point Likert scale (1: “highly unexpected”; 9: “highly expected”). Inferred uncertainty data resulted from computing the Shannon entropy of the distributions of expectedness ratings which had been normalised such that each distribution summed to unity (see [38] for details). Participants were explicitly instructed “not [to] think of the last note as the ultimate note of the melody, but rather as a continuation tone after which more notes may or may not come”. This addressed potential closure effects [62].

### 2.4. Analysis

The influence of specialised stylistic expertise was contrasted with that of general musical expertise using two statistical comparisons. Specifically, differences between jazz musicians and classical musicians were indicative of specialised expertise effects whereas differences between classical and non-musicians signified effects of generalised expertise. Overall, these contrasts were tested on four indices of expert predictive processing previously established by Hansen and Pearce [38]: (a) model-fit in terms of correlations between behavioural results and stylistically relevant model estimates; (b) condition effects on mean uncertainty consistent with the estimates of this model; (c) association between explicit and inferred measures of uncertainty; and (d) condition effects on mean expectedness, such that contexts with stylistically low entropy would lead to increased prediction error and thus lower expectedness on average than high-entropy contexts.

Before statistical testing, three types of data pre-processing took place. First, explicit certainty ratings were reversed to achieve consistency with the scale for inferred uncertainty. Thus, in the main analysis, this measure is referred to as explicit *un*certainty with 1 corresponding to “highly certain” and 9 to “highly uncertain”. Second, model estimates of probability were transformed into information content (IC) by taking the negative base-2 logarithm. This measure uses a more convenient scale than probabilities, has a clear interpretation in information theory as the number of bits required to encode an event in context [57], and relates to expectedness perceived by participants in listening experiments [38, 63]. Third, to assess correspondence between model estimates and behavioural responses, three bebop model-fit values and three general model-fit values were computed for each participant. These scores were the Pearson correlation between IC and expectedness ratings as well as between entropy and inferred and explicit uncertainty data. Due to the design of rating scales, positive model-fit manifested as negative correlations for expectedness and positive correlations for uncertainty.

The statistical testing proceeded as follows: For each of the three measures (i.e. expectedness, inferred uncertainty, and explicit uncertainty), one-way ANOVAs were conducted on individual model-fit values to assess expertise effects. Because these tests were conducted on separate dependent variables of interest, no correction for multiple comparisons was applied. Accompanying planned contrasts compared jazz vs. classical, classical vs. non-musicians, and jazz vs. non-musicians. Whereas the first two contrasts tested for effects of specialised and generalised expertise (as previously described), the third one was included for completeness.

Further, non-parametric correlation analysis investigated the relationship between model-fit values and composite jazz experience as well as the self-report measures and listening tests from *Goldsmiths Musical Sophistication Index* [59]. In statistically significant cases, multiple regression resolved whether specialised jazz experience explained unique variance not already explained by generalised expertise. Specifically, hierarchical regression was performed. The first step used a forward selection procedure where candidate predictors amongst the Gold-MSI measures were included one by one with the probability of *F* ≤ 0.05 as criterion. In the second step, jazz experience was included.

Mean expectedness and uncertainty were subjected to mixed 3x2 ANOVAs with expertise and condition as factors. Significant interactions justified paired-samples *t*-tests separately for each expertise group and one-way ANOVAs separately for the two conditions.

Finally, non-parametric correlations assessed the relationship between mean explicit and inferred uncertainty separately for each expertise group.

### 2.5. Ethics statement

The present study was approved by the Research Ethics Committee (QMREC 0954) at Queen Mary, University of London, and conducted according to the principles expressed in the Declaration of Helsinki. All participants provided prior written consent and received a compensation of £ 20 along with a detailed debriefing sheet upon completion of the study.

## 3. Results

### 3.1. Expectedness

**Fig 2:**
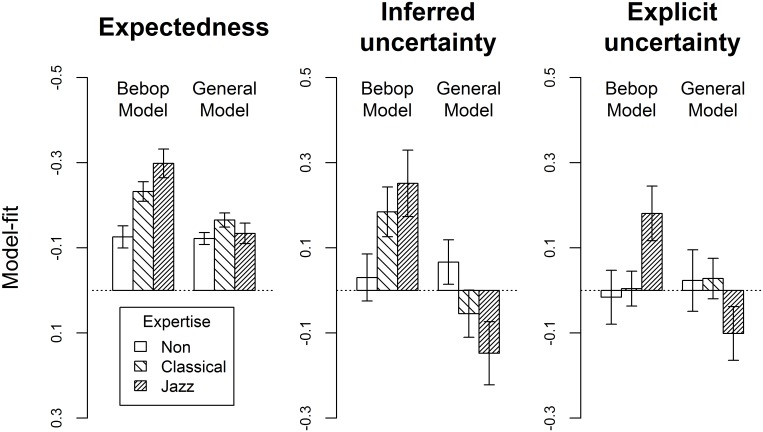
Model-fit. Mean correspondence between behavioural responses and computational model estimates (i.e. “model-fit”), plotted separately for non-musicians, classical musicians, and jazz musicians for two models trained on either bebop or general tonal music. Values positioned above the horizontal zero line designate good model correspondence whereas values below this line designate negative correspondence. For instance, jazz musicians perceive high levels of explicit uncertainty when entropy estimates of the bebop model are high whereas they perceive low uncertainty when the general model predicts high entropy. Error bars designate one standard error above and below the mean. Note that modest positive general model-fit for expectedness arises from high covariance of probability estimates from the two models. Similarly, artefactual negative general model-fit for explicit and inferred uncertainty results from actively ensuring a negative correlation between bebop and general entropy; importantly, this should not be ascribed to inverse following of the general model.

For the expectedness ratings, significant expertise effects were present for bebop model-fit, but remained absent for general model-fit (Fig 2, Table 2). Specifically, bebop model-fit was higher in musicians than in non-musicians whereas jazz and classical musicians did not differ significantly from one another (also see S3 Text for group-level analysis).

Bebop-model fit, furthermore, increased significantly with nearly all measures of general musical expertise represented by the subscales of the self-report questionnaire and the objective listening tests as well as with the composite measure of specialised jazz expertise (Table 3). None of these measures were associated with increases in general model-fit. Whereas a simple regression model only including the general musical training subscale explained 23% of the variance in bebop model-fit, *R*^*2*^ = .238, *Adj*. *R*^*2*^ = .225, *F*(1, 59) = 18.384, *p* < .001, there was a significant improvement, *F*(1, 58) = 4.599, *p* = .036, of adding jazz expertise as a second predictor, *R*^*2*^ = .294, *Adj*. *R*^*2*^ = .269, *F*(2, 58) = 12.052, *p* < .001 (see S3 Text for group-level analysis supporting this finding).

**Table 3:** Non-parametric correlations of model-fit and musical expertise.

		Gold-MSI: Self-report questionnaire						Gold-MSI: Tests		Jazz
		*F1*	*F2*	*F3*	*F4*	*F5*	*FG*	*Melodic memory (d’)*	*Genre sorting (adj*. *Rand index)*	*Composite jazz experience*
	Model-fit	*r*_*s*_*(62)*	*r*_*s*_*(62)*	*r*_*s*_*(62)*	*r*_*s*_*(62)*	*r*_*s*_*(62)*	*r*_*s*_*(62)*	*r*_*s*_*(62)*	*r*_*s*_*(62)*	*r*_*s*_*(62)*
**Bebop**	***Expectedness***	-.308*	-.253*	-.495***	-.196	-.360**	-.469***	-.394**	-.340**	-.527***
	***Inferred uncertainty***	.214	.133	.343**	.192	.264*	.351**	.297*	.263*	.361**
	***Explicit uncertainty***	.194	.059	.145	.019	.172	.202	.186	.212	.308*
**General**	***Expectedness***	.031	-.158	-.170	-.105	-.058	-.103	-.080	.188	-.124
	***Inferred uncertainty***	-.210	-.147	-.306*	-.197	-.223	-.326**	-.265*	-.303*	-.349**
	***Explicit uncertainty***	-.022	.032	-.058	.034	-.057	-.085	-.212	-.102	-.229

F1: Active Engagement; F2: Perceptual abilities; F3: Musical training; F4: Emotional response to music; F5: Singing abilities; FG: General musical sophistication.

* *p* < .050;

** *p* < .010;

*** *p* < .001.

**Fig 3:**
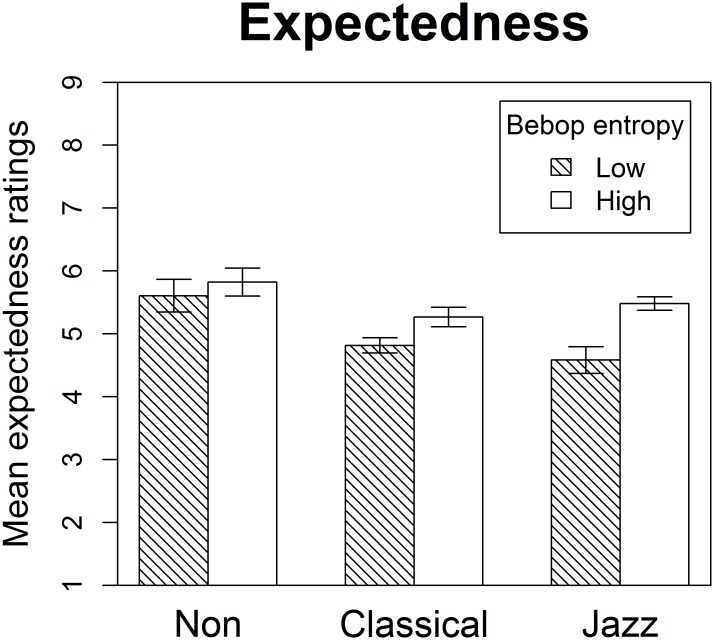
Mean expectedness. Mean expectedness ratings for non-musicians, classical musicians, and jazz musicians in the conditions with low and high degrees of bebop entropy. Stimuli in the low-bebop-entropy condition were simultaneously high in general entropy while stimuli in the high-bebop-entropy condition were simultaneously low in general entropy. Whereas the three groups of participants did not differ when bebop entropy was high, jazz and classical musicians experienced melodic continuations as more unexpected on average in the low-bebop-entropy condition. Error bars designate one standard error above and below the mean.

Expertise interacted significantly with condition for the mean expectedness ratings (Fig 3, Table 4). Whereas both jazz musicians and classical musicians perceived greater degrees of expectedness when bebop entropy was low, non-musicians’ ratings did not differ between the two experimental conditions. Moreover, significant expertise effects were only present in the condition with low bebop entropy, with both classical and jazz musicians differing significantly from non-musicians.

**Table 4:** Condition effects on mean expectedness, inferred, and explicit uncertainty.

			Expectedness^†^				Inferred uncertainty^α^				Explicit uncertainty			
**Test**	**Effect**		*F*	*df*	*p*	*η*^*2*^_*p*_	*F*	*df*	*p*	*η*^*2*^_*p*_	*F*	*df*	*p*	*η*^*2*^_*p*_
3x2 ANOVA	Expertise*Condition		6.49	2,58	.003**	.18	5.26	2,59	.008**	.15	2.85	2,59	.066	.09
	Expertise		-	-	-	-	-	-	-	-	1.89	2,59	.160	.06
	Condition		-	-	**-**	-	-	-	-	-	0.73	1,59	.398	.01
**Test**	**Subset**	**Effect**	*t*	*df*	*p*	*g*_*av*_	*t*	*df*	*p*	*g*_*av*_	*t*	*df*	*p*	*g*_*av*_
Paired-samples *t*-tests	Non	Condition	1.64	18	.119	0.27	0.10	19	.924	0.01	0.42	19	.683	0.08
	Classical	Condition	4.26	19	< .001***	0.70	3.12	19	.006**	0.44	0.73	19	.477	0.10
	Jazz	Condition	5.73	21	< .001***	1.10	3.39	21	.003**	0.65	2.09	21	.049*	0.50
**Test**	**Subset**	**Effect**	*F*	*df*	*p*	*η*^*2*^	*F*	*df*	*p*	*η*^*2*^	*F*	*df*	*p*	*η*^*2*^
One-way ANOVA	Low	Expertise	5.54	2,58	.006**	.16	4.38	2,59	.017*	.13	3.99	2,59	.024*	.12
	High	Expertise	1.90	2,58	.158	.06	0.79	2,59	.459	.03	0.28	2,59	.761	.01
**Test**	**Subset**	**Effect**	*t*	*Df*	*p*	*g*_*s*_	*t*	*df*	*p*	*g*_*s*_	*t*	*df*	*p*	*g*_*s*_
Independent-samples *t*-tests	Low	Jazz vs. class	0.91	40	.369	0.27	0.63	40	.531	0.19	2.73	40	.009**	0.83
	Low	Class vs. non	2.31	37	.025*	0.72	2.16	38	.035*	0.67	0.54	38	.593	-0.17
	Low	Jazz vs. non	3.25	39	.002**	1.00	2.84	40	.006**	0.86	2.10	40	.042*	0.64

* *p* < .050;

** *p* < .010;

*** *p* < .001.

^†^ One non-musician outlier was excluded from the mean expectedness data to ensure normality, as established by Shapiro-Wilk tests, all W ≥ 0.918, all p ≥ .069.

^α^ For inferred uncertainty, non-normality was present for jazz musicians in the high-bebop-entropy condition. See S4 Text for a demonstration of robust results after outlier exclusion.

### 3.2. Inferred uncertainty

For inferred uncertainty, the expertise effects for bebop and general model-fit both remained marginally non-significant (Fig 2, Table 2). Planned contrasts, however, suggested somewhat higher bebop model-fit in jazz-musicians compared to non-musicians. Bebop model-fit correlated significantly with three out of six Gold-MSI subscales and both listening tests as well as with specialised jazz expertise (Table 3). However, multiple regression analyses established that there was no significant advantage of adding jazz expertise, *F*(1, 59) = 1.280, *p* = .263, to the default model containing general musical training as the sole predictor, *R*^*2*^ = .142, *Adj*. *R*^*2*^ = .128, *F*(1, 60) = 9.950, *p* = .003. Due to the way that stimuli were selected based on difference scores between entropy estimates of the two computational models, significant negative correlations of musical expertise measures with general model-fit mirrored the significant positive correlations with bebop model-fit (Table 3).

**Fig 4:**
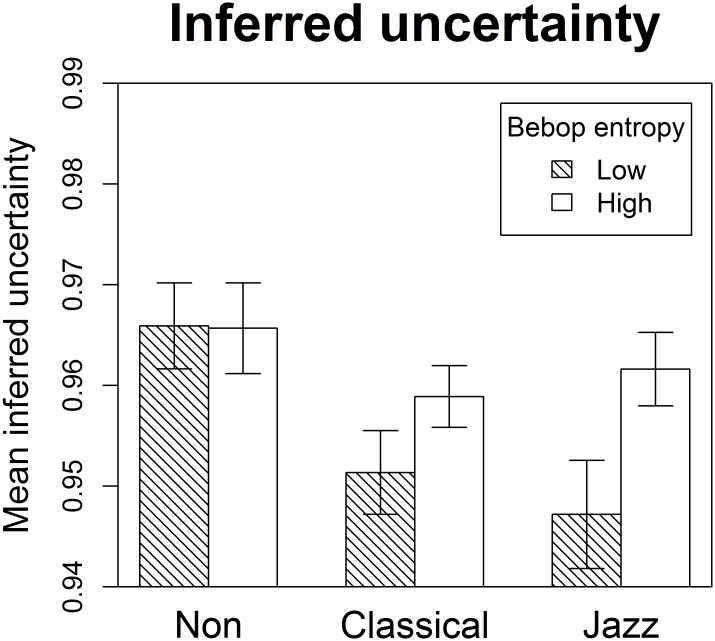
Mean inferred uncertainty. Mean inferred uncertainty for non-musicians, classical musicians, and jazz musicians in the conditions with low and high bebop entropy. Inferred uncertainty corresponds to the Shannon entropy of the distribution of expectedness ratings for each melodic context. Whereas non-musicians experienced similarly high degrees of uncertainty when exposed to melodies from either condition, jazz and classical musicians experienced lower degrees of uncertainty when entropy was estimated to be low according to the style-congruent bebop model. Error bars designate one standard error above and below the mean.

Turning to mean inferred uncertainty, a significant expertise-by-condition interaction was found, reflecting the fact that only jazz musicians and classical musicians experienced significantly greater inferred uncertainty in the condition with high bebop entropy (Fig 4, Table 4). This, in turn, led to significant expertise effects only for low-bebop-entropy stimuli where jazz musicians and classical musicians differed significantly from non-musicians.

### 3.3. Explicit uncertainty

For explicit uncertainty, model-fit with entropy estimates of the bebop model differed significantly between expertise levels with jazz musicians scoring higher than both classical and non-musicians (Fig 2, Table 2). No such expertise effects were present for general model-fit. Whereas measures of neither generalised nor specialised musical expertise correlated with general model-fit, only specialised jazz expertise correlated significantly with bebop model-fit (Table 3).

**Fig 5:**
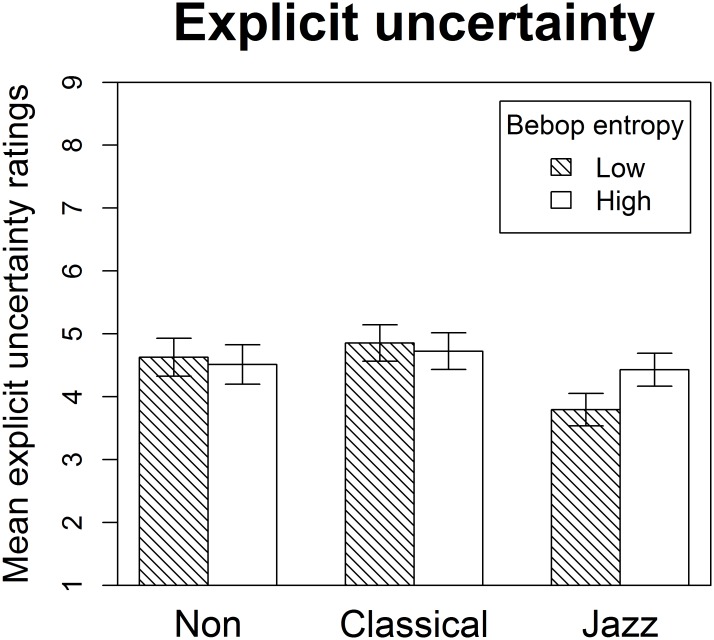
Mean explicit uncertainty. Mean explicit uncertainty for non-musicians, classical musicians, and jazz musicians in the two experimental conditions. The results mirror those for inferred uncertainty (see Fig 4), with the exception that classical musicians did not have explicit access to the implicit information that they had acquired about music in the bebop style. Error bars designate one standard error below and above the mean.

Unlike the similar analysis of expectedness and inferred uncertainty, expertise only interacted marginally non-significantly with condition effects on mean explicit uncertainty (Fig 5, Table 4). However, when this potential interaction was investigated further with planned contrasts, significant condition effects emerged in the expected direction specifically for jazz musicians, but remained absent for classical and non-musicians. Once again, significant expertise effects were only present for stimuli with low degrees of bebop entropy, but this time only jazz musicians differed significantly from non-musicians.

### 3.4. Relationship between explicit and inferred uncertainty

**Fig 6:**
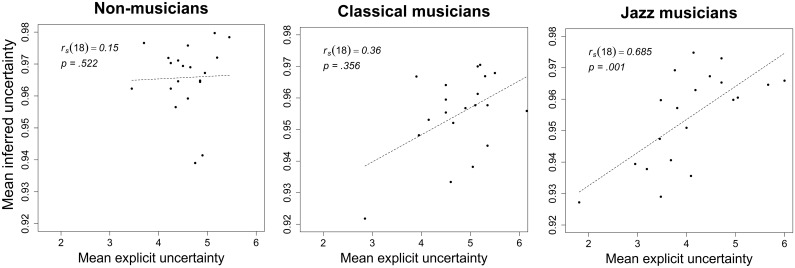
Mean explicit vs. inferred uncertainty. Scatterplots of the relationship between mean explicit and inferred uncertainty separately for the groups of non-musicians, classical musicians, and jazz musicians. This relationship was statistically significant for jazz musicians only.

The non-parametric correlation between mean explicit and mean inferred uncertainty averaged across all participants within each expertise group only reached significance for jazz musicians (Fig 6). This is consistent with the lack of model-fit with explicit uncertainty for classical and non-musicians demonstrated above.

## 4. Discussion

The present results demonstrate that highly specialised style-specific expertise influences explicit uncertainty processing. Specifically, only when probing uncertainty rather than expectedness and only when doing so with a method requiring explicit introspection did robust advantages of specialised expertise emerge. These effects manifested as significant differences in bebop model-fit between classical and jazz musicians and style-consistent effects of high and low entropy on mean explicit uncertainty selectively for jazz musicians. Additionally, only jazz-specific experience successfully explained significant proportions of the variance in bebop model-fit.

Moreover, significant correlations were found only for jazz-musicians between explicit uncertainty ratings and uncertainty inferred from the normalised distributions of expectedness ratings. This confirms that expertise increases the association between implicit and explicit (introspective) predictive processing [38]. This aligns well with bottom-up (implicit-to-explicit) models of skill learning according to which declarative knowledge develops from procedural knowledge [64]. These models draw support from reports that experts possess more abstract representations of musical styles that they have specialised in [24–26] and that explicit knowledge enhances discrimination [65]. Consolidating the importance of specialised training on expert predictive processing, the present results contribute a behavioural dimension to accumulating neuroscientific evidence for instrument- and genre-specific effects on auditory perception [13–20, 66, 67].

In contrast, for expectedness and inferred uncertainty, general expertise appeared to play a more prominent role than specialised expertise. The correlation of these two measures with bebop model estimates (i.e. bebop model-fit) was higher in musicians than in non-musicians whereas jazz and classical musicians did not differ significantly. Similarly, whereas non-musicians’ expectedness ratings were constant across conditions, classical and jazz musicians experienced contexts with low bebop entropy as less expected on average. This may be because stylistic experts experienced greater prediction error in low-entropy contexts [38]. Thus, when explicit introspection is assessed pertaining to the expectedness of musical events rather than to their prospective uncertainty, the remarkably strong human capacity for implicit statistical learning of regularities in the environment [68, 69] seems to compensate for the advantage of style-specific expertise. Importantly, however, general expertise was still significantly better than no expertise, thus disputing views that explicit training is not necessary for optimisation of predictive processing of music [70].

Despite the importance of generalised expertise, effect sizes were always numerically greater in jazz than in classical musicians. Particularly for expectedness, specialised expertise had marginally greater explanatory power. Similar conclusions were suggested by model-fit analysis on the group level (see S3 Text). Potentially, subtle effects of specialised expertise may be compromised by jazz musicians’ superiority in terms of listening skills. However, we believe this echoes characteristics of the population rather than the sample due to the perceived importance of these abilities in professional jazz practice [71].

In the present experiment, main instrument was selected to be representative of the genre rather than to be matched across groups. For this reason, there were more saxophone players amongst jazz than classical musicians. Future research should investigate whether stylistic effects on explicit predictive processing are mediated by instrument idiomatics, e.g. by comparing matched groups of jazz saxophonists and jazz non-saxophonists.

Moreover, generalised expertise effects could have been compromised by slightly higher jazz experience in classical than in non-musicians. Thus, while the results for expectedness and inferred uncertainty replicate Hansen and Pearce’s [38] findings for generalised expertise, they simultaneously call for replication with methods more suitable for revealing effects of specialised expertise.

Whereas classical and non-musicians’ predictive processing of bebop stimuli was somewhat less sophisticated than that of jazz musicians, they did not seem to be misapplying a stylistically irrelevant model. This mirrors previous observations that North-American listeners did not carry over Western pitch expectations when listening to Indian music [24]. Framing this question in a single cultural sphere with uniform pitch material, we can ascribe this finding to probabilistic properties rather than to artefacts of the musical material itself.

Along these lines, it has been argued that while acquisition of new knowledge (e.g. through statistical learning) can be adaptive for human survival, failure to limit its scope to relevant contexts may in itself cause dangerous or fatal situations [37]. Huron [33] has theorised this principle in terms of “cognitive firewalls” reserving the application of acquired probabilistic knowledge for specific circumstances. The biological disadvantage of misapplying an erroneous predictive model may explain why our non-musicians preferred an underdeveloped–and thus uncertain–bebop model over a well-developed general model. Though stylistically irrelevant, this latter model would at least have enabled high-certainty expectations to be formed.

It remains to be seen how listeners select a stylistically relevant model. We suggest that this process is cued by specific musical gestures, such as timbral cues enabling genre identification after a mere 250 ms [72]. Unsuccessful cue detection leading to irrelevant model misapplication has indeed been reported for twelve-tone music [22] and North-Sami yoiks [28]. Further work is needed to determine whether the use of bebop-specific listening schemas in the present study was prompted by saxophone timbre, swing rhythms, distinct pitch transitions, or a combination of these factors. Importantly, the present findings could be consolidated by demonstrating stylistic specialisation for classical sub-genres. While we expect that our two groups would in principle show reversed effects in such cases, high correlation between transitional statistics in classical genres and general tonal music may pose methodological challenges in this regard.

Summing up, as a novelty in music cognition research, we have distinguished the effects of stylistic expertise, thus diverting from generalised comparisons of musicians versus non-musicians. We cast this issue in terms of predictive processing [73], emphasising how schema-driven knowledge plays a crucial role in expert perception [29]. This perspective explains previous findings of more veridical guesses about melodic continuation in experts [28] in a probabilistic framework with reference to uncertainty processing [38]. Whereas earlier studies did not always control participants’ prior familiarity with specific musical stimuli [27, 28], we did so by focusing on improvised solos, which are stylistically constrained without promoting verbatim internalisation.

Additionally, by incorporating a computational model of expectation [53], we advocate a transition from using information theory for describing musical styles objectively (e.g. [56, 74] to using it for characterising listeners’ subjective perception [75]. Thus, we demonstrated that probabilistic properties of bebop jazz, which can be modelled with information theory and Markov chains, do not only characterise the generation of improvisation (see e.g. [35, 50, 52, 56]), but also its perception. This endorses a receptive rather than productive perspective on stylistic expertise.

While the present study focused on perceived uncertainty and expectedness, expertise-related differences in predictive processing are likely to underlie other established divergences between specialised and generalised experts. For instance, future research should explore whether more fine-grained segmentation skills [26] and better memory for pitch characteristics of novel home-culture music [76] in style-specific experts can be ascribed to more differentiated predictive processing. Similar accounts may explain why listeners sometimes perceive culturally familiar music as less tense [77] and less complex [78] than unfamiliar music and show greater accuracy in detecting intended emotions [79], mistunings [80], rhythmic deviations [81], and metric violations [82] in culturally familiar music. The last effect may, in turn, give rise to enhanced ability to perceive and tap in time with music on a wider range of levels within the metrical hierarchy [83]. Research on bimusicalism shows that home-culture bias in recognition memory and tension can be alleviated by enculturation in more than one musical tradition [77].

In conclusion, the current work demonstrates that stylistic enculturation explicates aspects of music perception and cognition that manifest specifically in terms of predictive uncertainty processing. Enhanced explicit processing appears to represent a key factor distinguishing specialised from generalised expertise. Thus, although expert jazz musicians may be better capable of characterising their musical genre of choice, they can of course always follow Louis Armstrong’s advice and refrain from doing so.

## Supporting Information

S1 Table Expectedness data. (XLSX)

S2 Table Inferred uncertainty data. (XLSX)

S3 Table Explicit uncertainty data. (XLSX)

S1 Text Stimulus selection. Further information on selection of stimuli.
(DOCX)

S2 Text Stimulus matching. Details on the matching of stimuli across the two experimental conditions.
(PDF)

S3 Text Bebop model-Fit on the group level. Further supporting analysis.
(PDF)

S4 Text Inferred uncertainty analysis excluding outliers. Supporting analysis of inferred uncertainty data excluding outliers.
(PDF)
