# Supplementary material for: "If You Have to Ask, You'll Never Know": Effects of Specialised Stylistic Expertise on Predictive Processing of Music
Source: PLoS One. 2016 Oct 12;11(10):e0163584. doi: 10.1371/journal.pone.0163584 (PMC5061385; doi:10.1371/journal.pone.0163584)
Supplement: S1 Text — Further information on selection of stimuli. (DOCX) [file pone.0163584.s004.docx]

S1 Text. Stimulus selection

The IDyOM model [1] was used to produce entropy estimates for stimulus selection. This computational model generates conditional probability distributions governing a pre-specified attribute of the next event at each consecutive point in a sequence, using unsupervised statistical learning and variable-order Markov modelling (for further details, see [1-4]. Each note event was represented by the model as a pair of values comprising pitch interval (i.e. the difference in semitones between consecutive notes) and scale degree (i.e. the interval in semitones from the tonal centre, as determined by the key signature in the Omnibook score).

Predictions from a short-term and a long-term sub-model were combined to produce a single probability distribution governing the pitch of the next note given the preceding context. Whereas the short-term sub-model always made predictions based on the current Charlie Parker melody or candidate context, crucially, the “bebop model “and “general model” differed in terms of prior training of their long-term sub-models. Specifically, the “general model” was trained on tonal folksongs, children’s songs, and hymns, originating from the *dva* (Deutsches Volksliedarchiv, http://www.dva.uni-freiburg.de/sammlungen/Deutsches_Volksliedarchiv) and *kinder* [5] subsets of the *Essen Folksong Collection* [6, 7] as well as *152 Nova-Scotian songs and ballads* [8] and the Church of England hymnal *Ancient and Modern* [9]. The “bebop model” used 10-fold cross-validation to make predictions about the Charlie Parker corpus based on subsets of the same corpus not containing the current target sequence.

Stimulus selection comprised an initial phase (“1st model run”) where 72 candidate contexts were selected from the target corpus and a second phase (“2nd model run”) where a subset of stimulus contexts was selected from the candidates (Fig A in S1 Text). In both phases, selection took place from the two tails of the distribution of events and contexts sorted according to difference scores between the entropy [10] of the probability distributions estimated by the two separate implementations of the computational model.


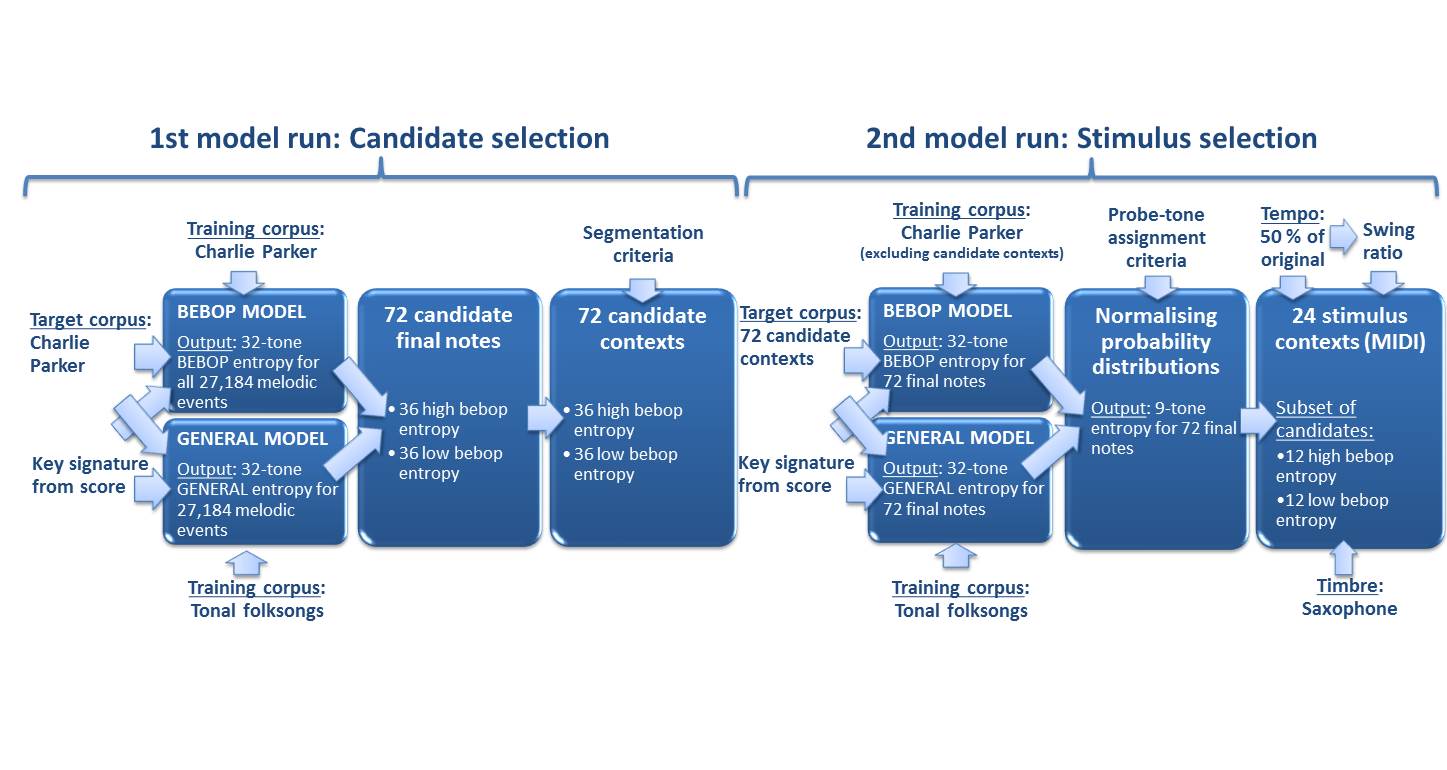


***Fig A.*** *Overview of the stimulus selection procedure: Two implementations of the computational model Information Dynamics of Music (IDyOM) were used to select 72 candidate contexts (1st model run) from which 24 stimuli were chosen (2nd model run). Whereas the “bebop model” was trained on Charlie Parker solos, the “general model” was trained on tonal folksongs and hymns. Note that contexts were selected based on the difference scores between bebop entropy and general entropy such that high-bebop-entropy contexts also had low general entropy and low-bebop-entropy contexts also had high general entropy (cf. Fig 1 in the main text).*

Seventy-two candidate notes were selected based on difference scores of entropy estimates from the first model run of the bebop and general models. Estimates relied on the full probability distributions for all 32 chromatically distributed pitches occurring in the training corpora (i.e. “32-tone entropy”). Candidate contexts were delimited by including context preceding the 72 candidate notes, following predetermined segmentation criteria. Specifically, all candidate contexts (1) contained at least 12 pitch events (before the probe tone), (2) contained at least six distinct pitches, (3) began with a note representing a phrase beginning in the original song, (4) did not contain parts of the song theme, and (5) did not overlap with other candidate contexts (i.e. to prevent contexts from providing information about actual continuations). Furthermore, (6) contexts in minor were excluded because reliability could not be guaranteed when only three songs in minor were contained in the corpus. Finally, (7) we excluded songs scoring higher than 2 on familiarity to the average jazz musician, as judged by two independent jazz experts on a 5-point Likert scale. This ensured the focus on schematic rather than veridical expectations (following Bharucha [11]; also see Huron [12, pp. 219-37] because verbatim memory of specific songs may produce low-uncertainty expectations irrespective of stylistic knowledge.

Nine probe tones were assigned to each candidate context, distributed a semitone apart and centred on the pre-probe-tone pitch. This procedure differed slightly from that of Hansen and Pearce [2] who centred the probe tones on the median pitch of the complete context; however, small, but non-negligible, effects of pre-probe-tone pitch height on inferred uncertainty in the previous study advised against repeating this procedure.

The two model implementations were subsequently run on candidate contexts rather than on the whole Omnibook corpus. Instead of 10-fold cross-validation, the bebop model was now trained on a modified version of the Omnibook corpus excluding notes from the candidate contexts. A subset constituting the final stimulus contexts were selected based on “9-tone entropy” computed from the normalised probability distributions governing the nine probe tones for each context.

Originally, each condition comprised 12 stimuli, but this number was reduced to 10 due to an encoding error in the candidate contexts. Specifically, stimulus selection was initially based on an erroneous key signature parameter for 76.4 % of the candidate contexts. However, after excluding four contexts which were slightly outside the intended quadrants, posthoc modelling using the correct parameter confirmed that the 20 final stimuli were within the intended range of difference scores (Fig B in S1 Text and Table A in S2 Text). Thus, data from these four contexts were excluded from all further analysis.

For both model runs, steps were taken to match the two training sets on size and pitch range. The Omnibook corpus originally contained 27,184 events spanning the 32-note pitch range D3-A5, but was reduced to 25,393 events after exclusion of events from the candidate contexts. To match this modified corpus as well as the original corpus reduced by 10 % due to the use of 10-fold cross-validation (i.e. 0.9 * 27,184 = 24,466 events), a random subset of 24,308 events from the previously described folksong, children’s song, and hymn corpus was selected as training set for the general model. Furthermore, the *dva* subset of the *Essen Folksong Collection* was transposed down four semitones to match the 32-note pitch alphabet.

| **1** | 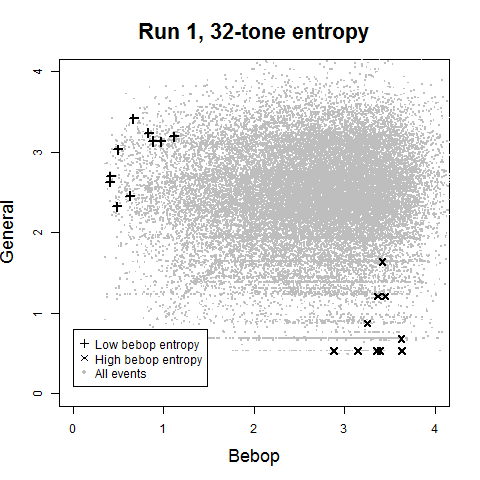 | **2** | 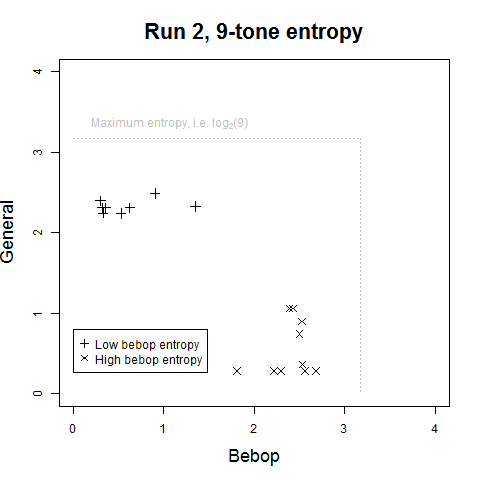 |
| --- | --- | --- | --- |

***Fig B.*** *Scatterplots of entropy estimates from the bebop and general models with the final 20 stimuli marked with + (low bebop entropy) and ⨉ (high bebop entropy). (1) The first model run pertained to the complete Charlie Parker corpus with the bebop model trained to that corpus (using 10-fold cross-validation) and the general model trained to tonal folksongs and hymns. Entropy computed from the full distribution of notes in the corpus (i.e. “32-tone entropy”) was used to select and segment the candidate contexts. (2) The second model run pertained to the 72 candidate contexts from which the final stimuli were selected based on the difference scores between the entropy estimates of the bebop and general models. For this selection process, “9-tone entropy” computed from a normalised probability distribution over the nine probe tones for each context was used.*

Due to a coding error, the key signature parameter was not changed accordingly causing erroneous scale degrees in ~25 % of the training set for the general model. However, all analysis reported in this paper is based on updated values using the correct key signatures. Very strong correlations between entropy estimates of the corrected and uncorrected models (first model run: *r* = .864; second model run: *r* = 0.920) establish that this only affected stimulus selection to a limited degree.

Consistent with the higher correspondence for the second model run, and because 32-tone entropy and information content can be used meaningfully to assess model correspondence across our three measures (i.e. expectedness, inferred uncertainty, and explicit uncertainty), all the analysis in the present paper uses 32-tone statistics from the second model run (rather than 32-tone statistics from the first model run or 9-tone statistics from the second model run). This is consistent with Hansen and Pearce (2014) (who used 37-tone statistics due to the slightly larger pitch alphabet in their training and test corpora). Fig 1 and Fig B in S1 Text show a highly consistent pattern of entropy estimates for the final stimuli.

***References:***

1. Pearce MT. The construction and evaluation of statistical models of melodic structure in music perception and composition. PhD Thesis, City University London, UK. 2005. Available: <http://doc.gold.ac.uk/~mas01mtp/papers/Pearce2005.pdf>
2. Hansen NC, Pearce MT. Predictive uncertainty in auditory sequence processing. Front Psychol. 2014;5: 1052. doi: 10.3389/fpsyg.2014.01052.
3. Pearce M, Ruiz MH, Kapasi S, Wiggins G, Bhattacharya J. Unsupervised statistical learning underpins computational, behavioural and neural manifestations of musical expectations. Neuroimage. 2010;50: 302-13.
4. Pearce MT, Wiggins GA. Auditory expectation: The information dynamics of music perception and cognition. Top Cogn Sci. 2012;4(4): 625-52.
5. Böhme FM. Deutsches Kinderlied und Kinderspiel: Volksüberlieferungen aus allen Landen deutscher Zunge. Wiesbaden, Germany: Breitkopf und Härtel; 1897.
6. Schaffrath H. The ESAC electronic songbooks. In: Hewlett WB, Selfridge-Field, E, editors. Computing in musicology: An international directory of applications, Vol. 9. Menlo Park, CA: Center for Computer Assisted Research in the Humanities; 1993. p. 78.
7. Schaffrath H. The ESAC databases and MAPPET software. In: Hewlett WB, Selfridge-Field, E, editors. Computing in musicology: An international directory of applications, Vol. 8. Menlo Park, CA: Center for Computer Assisted Research in the Humanities; 1992. p. 66.
8. Creighton H, editor. Songs and ballads from Nova Scotia. New York, NY: Dover; 1966.
9. Nicholson S, Knight GH, Dykes Bower J, editors. Ancient and Modern Revised. Suffolk, UK: William Clowes and Sons; 1950.
10. Shannon CE. A mathematical theory of communication. Bell Syst Tech J. 1948;27: 379-423, 623-56.
11. Bharucha JJ. Music cognition and perceptual facilitation: A connectionist framework. Music Percept. 1987;5(1).
12. Huron D. Sweet anticipation: Music and the psychology of expectation. Cambridge, MA: MIT Press; 2006.
