# Supplementary material for: "If You Have to Ask, You'll Never Know": Effects of Specialised Stylistic Expertise on Predictive Processing of Music
Source: PLoS One. 2016 Oct 12;11(10):e0163584. doi: 10.1371/journal.pone.0163584 (PMC5061385; doi:10.1371/journal.pone.0163584)
Supplement: S2 Text — Details on the matching of stimuli across the two experimental conditions. (PDF) [file pone.0163584.s005.pdf]

S2 Text. Stimulus matching

Stimuli in the two conditions were matched on musical features and information-theoretic properties to avoid confounding factors in the experimental manipulation (Table A in S2 Text). These were duration, metrical strength, and pitch height of the probe tone, musical key, tempo, and total number of events, and average entropy, information content, and median pitch height of the context as a whole. Stimuli were not fully matched on average bebop entropy, but this was not considered problematic given our experimental hypotheses.

Table A. Stimulus matching. Matching and differentiation of stimuli on musical features and information-theoretic properties estimated by the IDyOM model

		**Condition**		**Indep.-samples *t*-test**		**Mann-Whitney *U*-test**		**Chi-square test**	
		High bebop entropy	Low bebop entropy						
		M(sd)	*M(sd)*	*t(18)*	*p*	*U(20)*	*p*	$\chi^2(5)$	*p*
Probe-tone	Duration	9.5 (3.4)	10.0 (3.3)	0.33	.743				
	Metrical strength	-2.4 (1.4)	-1.7 (1.2)	1.24	.229				
	Pitch height	64.6 (3.3)	65.5 (6.1)			39	.436		
	Bebop entropy ②, 9t	2.39 (0.08)	0.74 (0.14)			0	<.001		
	General entropy ②, 9t	0.55 (0.11)	2.33 (0.02)			0	<.001		
	Bebop entropy ②, 32t	3.34 (0.13)	0.98 (0.17)			0	<.001		
	General entropy ②, 32t	0.83 (0.12)	2.97 (0.06)			0	<.001		
	Abs entropy difference ①, 32t	2.53 (0.41)	2.24 (0.29)			29	.123		
	Abs entropy difference ②, 9t	1.84 (0.38)	1.59 (0.45)			35	.280		
	Abs entropy difference ②, 32t	2.52 (0.48)	2.00 (0.44)			21	.029		
Context	Key signature	-	-					8.28	.141
	Number of events	25.3 (11.0)	21.5 (6.1)	-0.96	.351				
	Notated tempo	225 (88.9)	266 (51.8)	1.25	.229				
	Median pitch height	67.4 (3.1)	67.5 (3.7)	0.07	.949				
	Mean Bebop entropy ②, 32t	2.79 (0.33)	2.52 (0.15)	-3.24	.005				
	Mean General entropy ②, 32t	2.37 (0.14)	2.43 (0.14)	0.88	.391				
	Mean Bebop IC ②, 32t	2.89 (0.77)	2.33 (0.49)	-1.95	.067				
	Mean General IC ②, 32t	4.57 (1.00)	4.86 (0.63)	0.79	.438				
Pre-probe-tone	Bebop IC ②, 32t	3.98 (0.70)	3.00 (0.91)			35	.280		
	General IC ②, 32t	6.49 (0.78)	7.03 (0.85)			42	.579		

①: First model run; ②: Second model run; 9t: 9-tone entropy; 32t: 32-tone entropy.
