# Supplementary material for: "If You Have to Ask, You'll Never Know": Effects of Specialised Stylistic Expertise on Predictive Processing of Music
Source: PLoS One. 2016 Oct 12;11(10):e0163584. doi: 10.1371/journal.pone.0163584 (PMC5061385; doi:10.1371/journal.pone.0163584)
Supplement: S3 Text — Further supporting analysis. (PDF) [file pone.0163584.s006.pdf]

S3 Text. Bebop model-fit on the group level

Further non-parametric model-fit analysis was carried out on the group level to validate the results relating to individual model-fit reported in the main text above. Specifically, separately for the three expertise levels, mean expectedness ratings for each context were correlated with information content (IC) as estimated by the bebop and general models.

For the stylistically relevant bebop model, significant expectedness model-fit was present on the group level for all three expertise groups (Fig A in S3 Text). Notably stronger effect sizes were present on the group level compared to individual model-fit values (Fig 2 in the main text). William’s *t*-test of dependent correlations [1] established greater bebop model-fit in classical musicians compared to non-musicians, $t(177) = 2.841$, $p = .005$. Jazz musicians further exceeded classical musicians, $t(177) = 2.852$, $p = .005$. When comparing bebop model-fit with general model-fit directly, significant differences were found for jazz musicians, $t(177) = 3.833$, $p < .001$, whereas expectedness ratings provided by classical musicians, $t(177) = 1.634$, $p = .104$, and non-musicians, $t(177) = 0.376$, $p = .707$, were not better predicted by bebop than by general IC.

Hence, the results of the main analysis were replicated on the group level with even greater effect sizes, demonstrating levels of stylistic learning in classical musicians that was both distinctly higher than non-musicians and distinctly lower than jazz musicians. This gives important nuance to the main analysis where classical and jazz musicians were not statistically distinguishable on bebop model-fit for expectedness ratings.

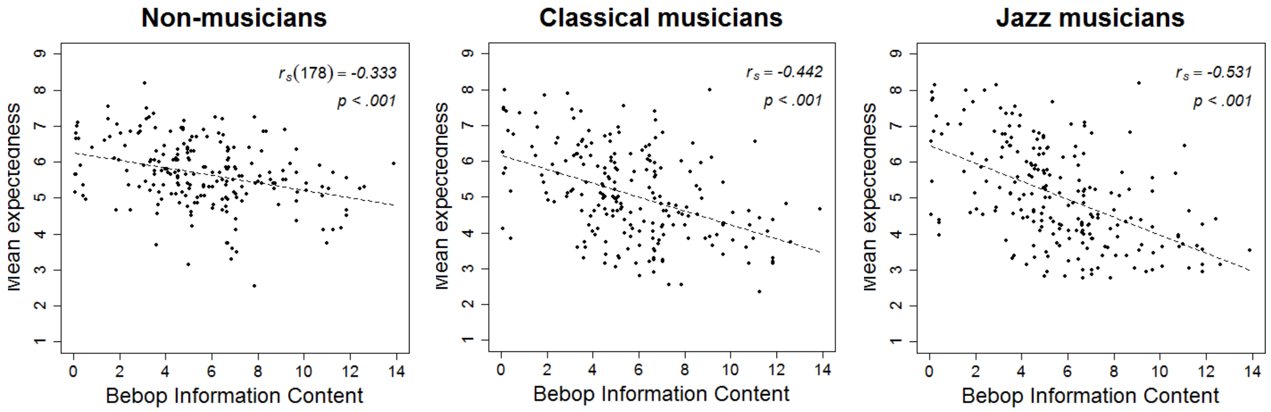

Fig A. Group-level bebop model-fit for expectedness ratings. *This is expressed as mean expectedness ratings for each context plotted against information content as estimated by the bebop model separately for non-musicians, classical musicians, and jazz musicians. As evident, this linear relationship is significant for all three groups, but increases significantly from non-musicians to classical musicians and from classical musicians to jazz musicians.*

References:

1. Steiger JH. Tests for comparing elements of a correlation matrix. Psychol Bull. 1980;87(2): 245-51. doi: 10.1037/0033-2909.87.2.245.
