# Supplementary material for: "If You Have to Ask, You'll Never Know": Effects of Specialised Stylistic Expertise on Predictive Processing of Music
Source: PLoS One. 2016 Oct 12;11(10):e0163584. doi: 10.1371/journal.pone.0163584 (PMC5061385; doi:10.1371/journal.pone.0163584)
Supplement: S4 Text — Supporting analysis of inferred uncertainty data excluding outliers. (PDF) [file pone.0163584.s007.pdf]

S4 Text. Inferred uncertainty analysis excluding outliers

Prior to analysis of mean inferred uncertainty data, Shapiro-Wilk's test showed non-normality in the high bebop entropy condition specifically for jazz musicians, $W(20) = 0.846$, p = 003. Due to slight bimodality caused by four outliers grouped close together, normality could only be obtained when excluding all four jazz musicians, $W(18) = 0.968$, p = 982. When doing so and re-running the analysis reported above (i.e. 3x2 mixed ANOVA and separate paired-samples t-tests for each expertise group), the findings remained fully robust with both a significant expertise-by-condition interaction, $F(2, 55) = 6.627$, $p = .003$, $\eta^2_p = .194$, and significant differences in inferred uncertainty between the two conditions for jazz musicians, $t(17) = 3.586$, $p = .002$. Similarly, expertise effects were significant in the condition with low bebop entropy, $F(2,55) = 3.177$, $p = .049$, but remained non-significant for high-bebop-entropy stimuli, $F(2,55) = 2.179$, $p = .124$.
